# Relationship of widowhood with pulse pressure, fasting blood glucose, and mental health in older adults: a propensity matching score analysis

**DOI:** 10.3389/fpubh.2023.1257133

**Published:** 2023-10-25

**Authors:** Yi Zhang, Xiangfan Chen, Yimei Sun, Sujuan Feng, Fang Wang, Haiyan Gu, Hanyu Jia, Quanxing Zhang, Wenbin Ding, Hongjian Lu, Jidong Zhang

**Affiliations:** ^1^Department of Science and Education, Nantong First People's Hospital, Nantong, China; ^2^Department of Biobank, Nantong First People's Hospital, Nantong, China; ^3^Finance Department, Nantong First People's Hospital, Nantong, China; ^4^Blood Dialysis Room of Nantong First People’s Hospital, Nantong, China; ^5^Nursing Department of Nantong First People’s Hospital, Nantong, China; ^6^The President's Office, Nantong First People's Hospital, Nantong, China

**Keywords:** older adults, widowhood, pulse pressure, fasting blood glucose, mental health

## Abstract

**Background:**

Transitioning from marriage to widowhood presents inevitable and significant challenges for many older adults. This study explored the impact of widowhood on a range of mental health outcomes, including pulse pressure and fasting blood glucose levels, among older adults in nursing homes.

**Methods:**

This cross-sectional study utilized cluster random sampling to recruit participants, with data analyzed from 388 older Chinese adults. Psychosocial traits were assessed using the Perceived Social Support from Family scale (PSS-Fa) for family support, the Generalized Anxiety Disorder 7-item scale (GAD-7) for anxiety symptoms, and the 9-item Patient Health Questionnaire (PHQ-9) for depressive symptoms and suicidal ideation. Propensity score matching (PSM) was employed to control for confounding factors. A multivariate linear regression analysis was performed to explore the relationship between widowhood, mental health outcomes, pulse pressure, and fasting blood glucose levels.

**Results:**

After applying PSM, the sample size was refined to 268 (*N* = 134 for both married and widowed groups) from the initial 388, excluding 120 unmatched cases. Widowed older adults were found to have notably lower family support (*β* = −0.81, *p* = 0.002), increased depressive symptoms (*β* = 1.04, *p* = 0.043), elevated pulse pressure (*β* = 8.90, *p* < 0.001), and higher fasting blood glucose levels (*β* = 3.22, *p* = 0.027). These associations exhibited greater beta values compared to pre-matching analysis.

**Conclusion:**

Our findings revealed that widowed participants had reduced family support, an increased risk of depressive symptoms, heightened pulse pressure, and elevated fasting blood glucose in comparison to their married counterparts. Interventions focusing on social support, mental health, and cardiovascular well-being could be advantageous for this at-risk group.

## Introduction

In recent years, a noticeable global trend of population aging has emerged, underpinned by advances in longevity and declining mortality rates (1). By 2030, it’s projected that over 1 billion individuals globally will be classified as older adults (2). Guo’s research suggests that China will witness a surge in its aging population by 2050, where those aged 60 and above will constitute approximately a quarter of its total populace (3). This demographic shift is primarily due to China’s one-child policy (4) coupled with the contemporary aspirations of younger couples prioritizing education and professional growth (5). Consequently, many families grapple with the challenge of adequately caring for their older adults loved ones, leading a significant portion of this aging population to opt for nursing home residency (6). However, it’s concerning to note that individuals in nursing homes tend to report higher instances of psychological distress than their counterparts living within family homes, often stemming from a lack of autonomy and the absence of close familial bonds (7). Recognizing and proactively addressing these issues is paramount to fostering a holistic, supportive environment in nursing homes, which is crucial for the overall well-being and mental health of older adults.

Widowhood stands out as a substantial determinant influencing the health of older individuals (8, 9). As couples age, the probability of encountering widowhood naturally increases. Data from a 2000 census spanning several countries, including the United Kingdom, Italy, Finland, France, Greece, Australia, and Russia, has revealed that the proportion of widowed older adults women fluctuates between 52.8% and 65% (10). Drawing from the Sixth National Population Census of China, it’s discerned that the widowed older adults demographic in China amounts to 47.74 million, representing 26.89% of the country’s total older adults population. As the dynamics of an aging society continue to evolve, projections suggest that by 2050, the number of widowed individuals in China can soar to an astounding 118.4 million (11).

Transitioning from marriage to widowhood is an inescapable and formidable journey that many older adults endure. In later life, widows and widowers emerge as a particularly susceptible group burdened by the profound stress of bereavement. This can manifest in intense grief and financial strains (12). A wealth of research indicates that widowhood correlates with a heightened susceptibility to various health complications, encompassing illnesses, mental health disturbances, disability, and even increased mortality (13–15). A community-oriented cross-sectional survey from China has found that widowed individuals are more prone to manifest symptoms of somatization and phobic anxiety (16). Echoing this, Amato’s Divorce-Stress-Adjustment Perspective elucidates that widowhood ushers in a multitude of stressors, intensifying the probability of emotional, behavioral, and health afflictions (17). Given this context, it becomes imperative for society at large, as well as policymakers and institutions, to champion the mental health and holistic well-being of China’s older adults.

Historically, the relationship between diverse variables in research has largely been scrutinized using linear or logistic regression models. Yet, in comparison to randomized controlled trials (RCTs), these models offer only a modicum of control over confounding elements. Addressing this gap, recent methodologies advocate for propensity score matching (PSM) analysis (18, 19). PSM aims to equilibrate covariate distributions by segmenting samples into treatment and control cohorts, allocating propensity scores grounded in initial socio-demographic variables (20). Subsequent to this, unmatched samples are discarded, retaining matched samples for the final analytical phase. Despite its potential, the adoption of PSM as an analytical tool remains scant.

In the existing literature, the focus has predominantly converged on the repercussions of widowhood on mental health dimensions, such as depressive manifestations or suicidal tendencies in the older adults (14, 21, 22). Yet, a discernible research void persists regarding widowhood’s bearing on facets like family support. Concurrently, there’s a noticeable dearth of studies probing the nexus between widowhood and parameters like pulse pressure (PP) and fasting blood glucose, particularly among older Chinese individuals. These metrics are pivotal; elevated PP not only taxes the heart, heightening the susceptibility to cardiovascular incidents but also fosters arterial degradation, amplifying atherosclerotic risks (23, 24). Fasting blood glucose, a barometer of blood sugar post a fasting interval, is instrumental in diabetes risk evaluation. Ascendant levels portend an augmented risk of diabetes onset and cardiovascular complications (25, 26).

In this study, we aimed to achieve several specific goals. First, we explored the effect of widowhood on mental health, especially regarding family support, among older adults in nursing homes. Second, we examined how widowhood affected physiological indices, including PP and fasting blood glucose levels. Finally, we evaluated the efficacy of PSM in controlling for confounders by comparing outcome differences between the pre-PSM and post-matching stages.

## Materials and methods

### Participants and procedures

We conducted a cross-sectional study using cluster random sampling to assess older adults in nursing homes. Based on economic classifications within Nantong City, four nursing homes were randomly selected as our survey sites. From March 3 to March 25, 2023, participants were recruited from the following nursing homes in Nantong City, Jiangsu Province, China: Nantong Yincheng Health Care Hospital, Nantong Sunshine Nursing Home, OlderYiyuan, and Nantong Sunshine New City Rehabilitation Nursing Home.

Our survey utilized the Wenjuanxing Software, a prominent online questionnaire platform. A trained researcher elucidated the study’s purpose to each participant before distributing our questionnaire link. Emphasizing anonymity, participants were encouraged to fill out the questionnaire autonomously, with the freedom to contact our researchers for any survey-related questions. Participants were also informed of their rights, including the option to withdraw from the survey at any point. Typically, it took most participants around 15 min to complete the questionnaire.

Eligible participants for the survey were those aged 60 years or older, residing in one of the four selected nursing homes, without major mental illnesses, and who were willing to provide written informed consent. Conversely, individuals who felt overwhelmed by the questionnaire and opted to discontinue midway or did not disclose their marital status were excluded. The cross-sectional survey formula was applied to calculate the sampling as follows:


$$N=\frac{Z_{1-\partial /2}^{2}\times pq}{d^{2}}$$

*Z*_1−*∂*/2_ is the statistical value for significance testing, where *α* = 0.05 and its value is 1.96. *p* is the prevalence rate of outcome variables, where *q* = 1−*p* and *d* is the allowable error, where *d* = 0.2*p*. Prior research indicates that the prevalence rate of outcome variables in older adults spans between 26.6% and 48.0% (27, 28). For our calculations in this study, we used the lower threshold of 26.6%, necessitating a sample size of at least 292 subjects when accounting for a 10% non-response rate.

Of the 602 older individuals surveyed, 506 questionnaires were fully completed, reflecting a response rate of 84.1%. Importantly, 118 older adults did not specify their marital status. Given our study’s focus on married and widowed individuals, these respondents were excluded from the final analysis. Consequently, our analysis encompassed 388 older adults: 239 married and 149 widowed. The average age of this cohort was 83.97 years, with a standard deviation (SD) of 7.19 years.

Ethical approval for this study was granted by the Ethics Committee of Nantong First People’s Hospital under the identification number 2023KT091.

### Measures

#### Basic socio-demographic variables

We gathered essential socio-demographic information from participants, which included age, sex, ethnicity, marital status, residence, monthly income, and educational level. Additionally, we measured the height and weight of each participant to determine their body mass index (BMI) [BMI = Weight (kg) /Height^2^ (m^2^)]. The BMI scores were then categorized into four groups: underweight (< 18.5), normal (18.5–24.9), overweight (25.0–29.9), and obese (≥ 30.0).

#### Family support

Family support was gauged using the Perceived Social Support from Family scale (PSS-Fa) (29). This scale consists of 15 items, each requiring a response of “yes” (scored as 1) or “no” (scored as 0). The overall score can range between 0 and 15, where a higher score signifies greater perceived family support. In our study, the PSS-Fa demonstrated good internal consistency, reflected by Cronbach’s *α* coefficient of 0.75.

#### Anxiety symptoms

To evaluate anxiety symptoms, we employed the Generalized Anxiety Disorder 7-item scale (GAD-7). This scale consists of seven items, with participants responding to each on a four-point Likert scale: from “not at all” (scored as 0) to “nearly every day” (scored as 3). By summing the scores of individual items, total scores can range from 0 to 27, where higher scores denote more pronounced anxiety symptoms. In this study, the GAD-7 exhibited excellent internal consistency, as evidenced by Cronbach’s *α* coefficient of 0.94.

#### Depressive symptoms

We used the 9-item Patient Health Questionnaire (PHQ-9), developed by Kroenke et al. (30), to assess depressive symptoms. This questionnaire features nine items, and participants indicate the frequency of each symptom over the past 2 weeks, ranging from “not at all” (scored 0) to “nearly every day” (scored 3). The PHQ-9’s total score, which can vary from 0 to 27, is obtained by summing individual item scores, with higher values indicating more severe depressive symptoms. In our study, the PHQ-9 exhibited excellent internal consistency, reflected by Cronbach’s *α* coefficient of 0.91.

#### PP and fasting blood glucose

In this study, systolic blood pressure (SBP) and diastolic blood pressure (DBP) were measured for participants seated after resting for at least 5 min using a mercury column sphygmomanometer. PP was calculated as the difference between SBP and DBP, with the formula PP = SBP − DBP. Fasting blood glucose levels adhered to WHO recommendations, taken after an 8–12 h overnight fast using an Accu-Chek Performa glucometer. Participants were informed about the study during their routine care, ensuring they were prepared for the fasting requirement.

### Statistical analysis

#### Descriptive analysis

Descriptive analyses expressed qualitative data as numbers and percentages (*N*/%), while quantitative data were presented as mean ± SD. To examine differences in basic socio-demographic variables based on marital status, we employed the chi-square test and *t*-test.

#### PSM analysis

To account for potential confounders, we employed PSM analysis to align baseline socio-demographic characteristics, including age, sex, residency, education level, monthly income, and BMI. In our study, the experimental group comprised married individuals, whereas the control group consisted of widowed individuals. We implemented nearest neighbor matching with a caliper set at 0.05 and maintained a 1:1 ratio for cases to controls.

#### Multivariate linear regression

We employed multivariate linear regression models to evaluate the effect of marital status on mental health outcomes, PP, and fasting blood glucose, both pre- and post-matching. The models also incorporated basic socio-demographic covariates. In these analyses, widowhood served as the independent variable (*x*-variable), while the dependent variables (*y*-variables) were mental health outcomes, PP, and fasting blood glucose.

For PSM analysis, we used R version 4.0.2. All other statistical analyses were conducted using SPSS version 21.0. A *p*-value of 0.05 (two-tailed) was the threshold for statistical significance in this study.

## Results

### General characteristics of older adults

As shown in Table 1, all participants belonged to the Han ethnicity. The sample included 242 females, accounting for 62.4% of the cohort. A majority, 298 older individuals (76.6%), were married. Furthermore, 117 participants (30.2%) reported a monthly disposable income in the range of 3,001–6,000 yuan. Notably, 127 (32.7%) of these older adults presented with varying levels of BMI abnormalities while residing in Chinese nursing homes.

**Table 1:** Socio-demographic characteristics and key variables outcomes of participants (*N* = 388).

Characteristic	Number	Percent
*Age (years)*	83.97 ± 7.19	
60–69	12	3.1
70–79	77	19.8
80–89	219	56.5
90–100	80	20.6
*Sex*		
Women	242	62.4
Men	146	37.6
*Ethnic*		
Han	388	100.0
*Marital status*		
Married	239	61.6
Widowed	149	38.4
*Residence*		
Rural	45	11.6
Urban	343	88.4
*Education level*		
High school or lower	281	72.4
Bachelor’s degree or above	107	27.6
*Income (monthly)*		
500 or lower	34	8.8
501–1,000	51	13.1
1,001–3,000	79	20.4
3,001–6,000	117	30.2
6,001–9,000	58	14.9
9,001 or higher	49	12.6
*BMI*	22.11 ± 3.14	
Low weight	36	9.3
Normal weight	261	67.3
Overweight	75	19.3
Obesity	16	4.1

### The performance of PSM analysis and its outcomes

**Figure 1:**
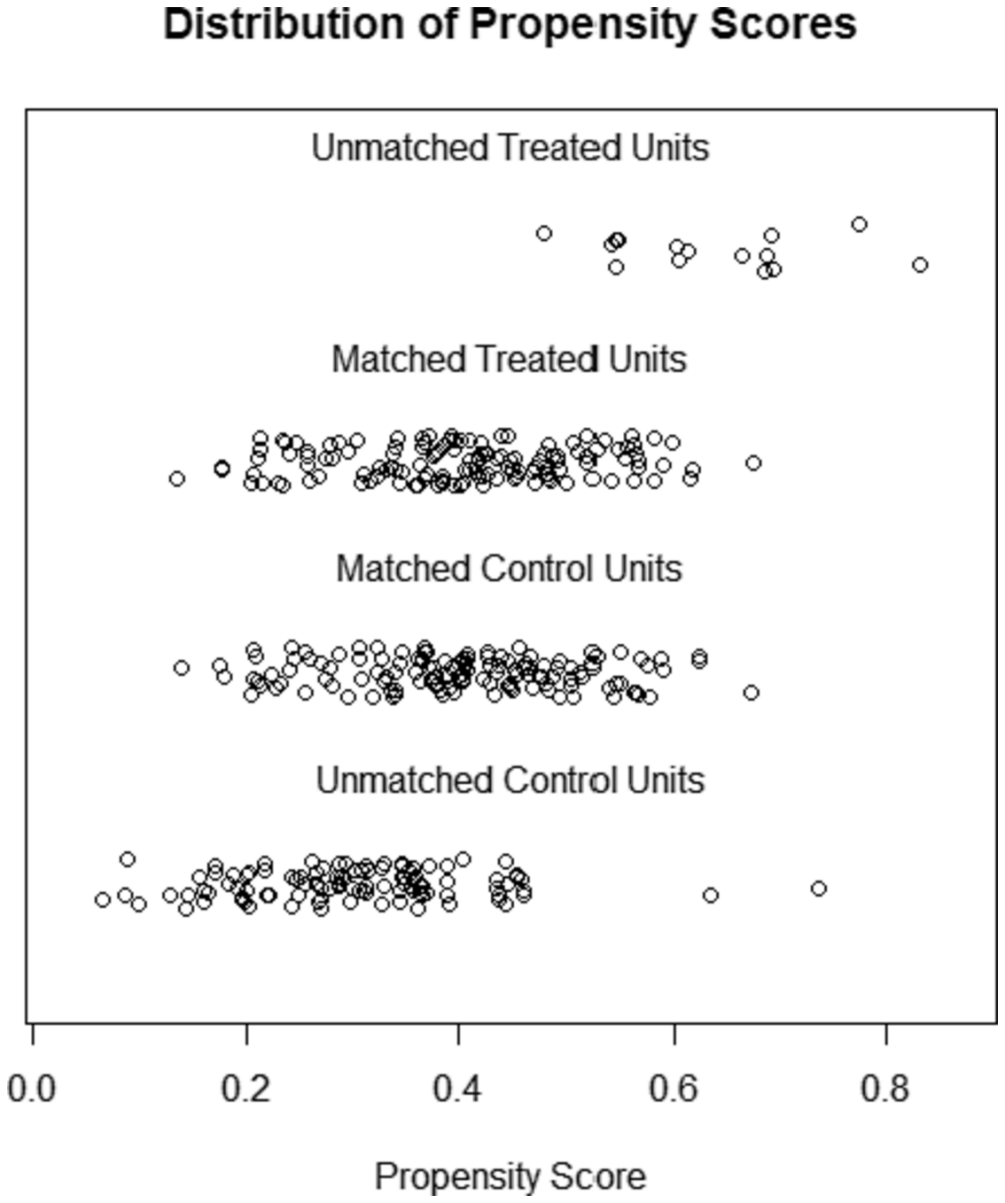
The distribution of propensity score before and after PSM analysis.

In our PSM analysis, we designated married older individuals as the control group and compared them with their widowed counterparts in the treatment group. Figure 1 illustrates the propensity score distributions for both unmatched and matched samples. Notably, the total sample size diminished from 388 to 268, with 134 individuals each in the married and widowed categories, leading to the exclusion of 120 unmatched cases.

**Figure 2:**
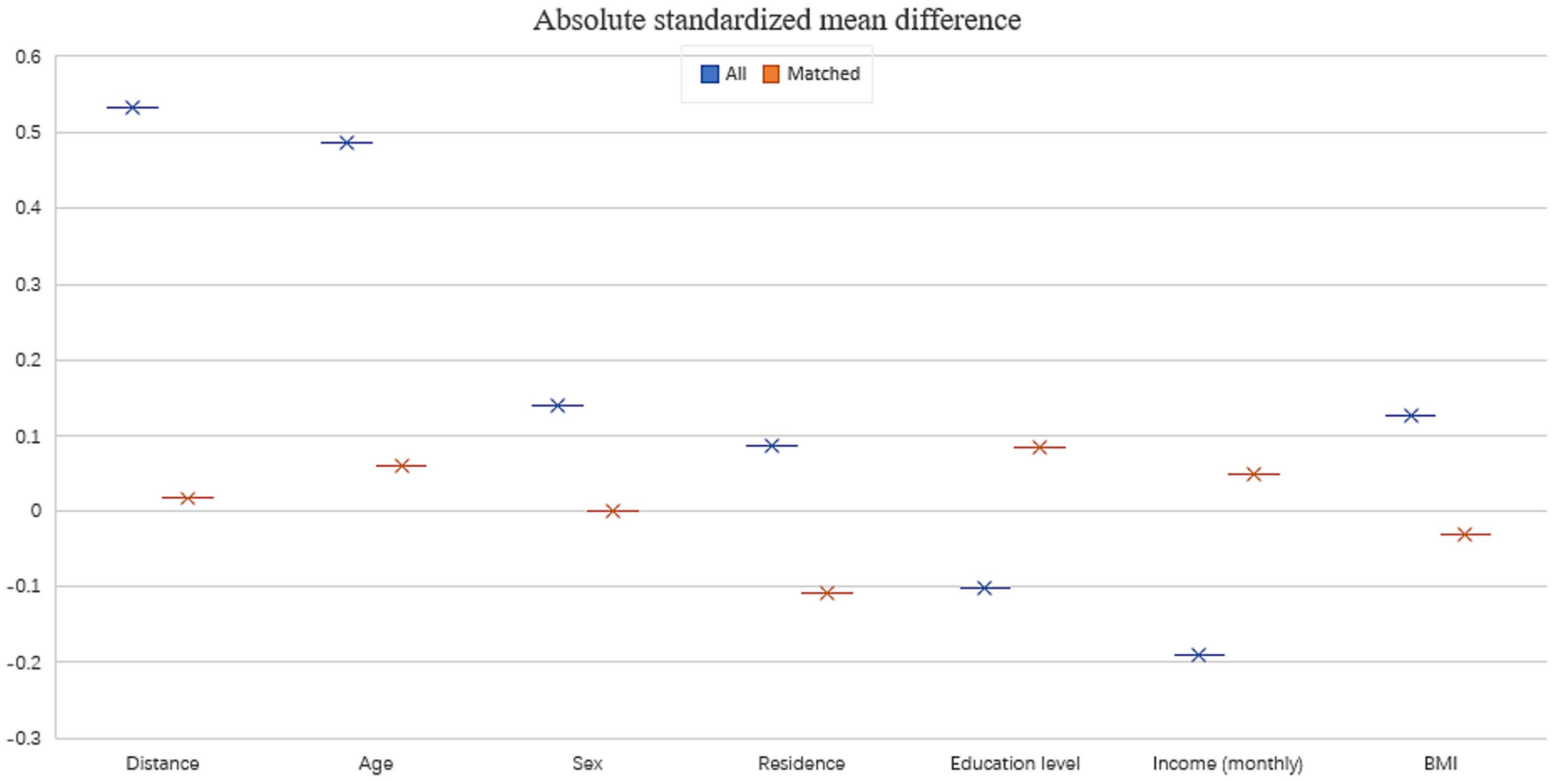
The absolute standardized mean difference before and after PSM analysis.

Before matching, the basic socio-demographic characteristics had an absolute standardized mean difference of 0.533. This difference decreased substantially to 0.016 after matching, indicating an excellent covariate balance in the post-matching samples (Figure 2). Additionally, chi-square and t-test results for these socio-demographic characteristics, categorized by marital status both pre- and post-matching, further validated the effectiveness of our PSM analysis. This efficacy was evidenced by the notable reduction in chi-square/*T*-value and *p*-value (Table 2).

**Table 2:** The socio-demographic characteristics of participants before and after PSM between married and widowed older adults.

Variables	Before PSM analysis (*N* = 388)		*χ*^2^	*p*	After PSM analysis (*N* = 268)		*χ*^2^	*p*
	Married (*N* = 239)	Widowed (*N* = 149)			Married (*N* = 134)	Widowed (*N* = 134)		
Sex			1.71	0.191			0.001	0.999
Women	143 (59.8)	99 (66.4)			48 (35.8)	48 (35.8)		
Men	96 (40.2)	50 (33.6)			86 (64.2)	86 (64.2)		
Ethnic			–	–			–	–
Han	239 (100.0)	149 (100.0)			134 (100.0)	134 (100.0)		
Residence			0.79	0.375			0.75	0.386
Rural	25 (10.5)	20 (13.4)			22 (16.4)	17 (12.7)		
Urban	214 (89.5)	129 (86.6)			112 (83.6)	117 (87.3)		
Education level			0.91	0.339			0.51	0.476
High school or lower	169 (70.7)	112 (75.2)			104 (77.6)	99 (73.9)		
Bachelor’s degree or above	70 (29.3)	37 (24.8)			30 (22.4)	35 (26.1)		
Income (monthly)			35.62	<0.001			22.29	<0.001
500 or lower	10 (4.2)	24 (16.1)			8 (6.0)	19 (14.2)		
501–1,000	39 (16.3)	12 (8.1)			25 (18.7)	10 (7.5)		
1,001–3,000	56 (23.4)	23 (15.4)			37 (27.6)	20 (14.9)		
3,001–6,000	57 (23.8)	60 (40.3)			33 (24.6)	55 (41.0)		
9,001 or higher	35 (14.6)	16 (10.7)			18 (13.4)	14 (10.4)		
	Mean ± SD	Mean ± SD	*T*	*p*	Mean ± SD	Mean ± SD	*T*	*p*
Age	82.74 ± 7.31	85.93 ± 6.55	−4.35	<0.001	84.86 ± 6.47	85.27 ± 6.47	−0.51	0.611
BMI	21.94 ± 2.98	22.37 ± 3.38	−1.32	0.188	22.03 ± 3.29	21.93 ± 2.83	0.28	0.783

### The relationship between marital status and mental health outcomes, PP, and fasting blood glucose

**Figure 3:**
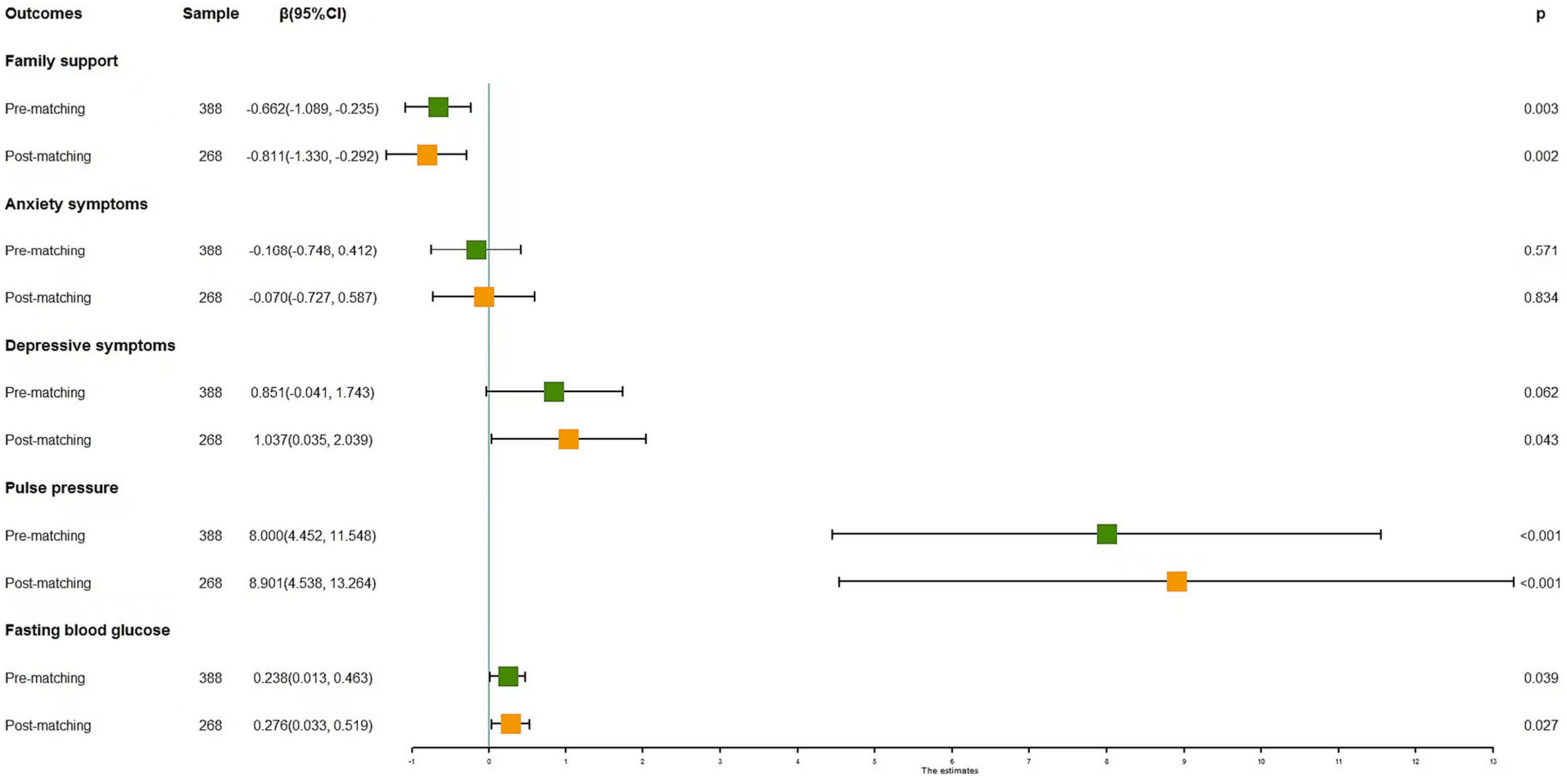
The effect of widowhood on PP, fasting blood glucose, and mental health in older adults before and after PSM analysis.

Figure 3 delineates the interplay between marital status and the outcomes of mental health, PP, and fasting blood glucose among older residents of Chinese nursing homes, both prior to and subsequent to the matching process. In the pre-matching phase, after adjusting for socio-demographic covariates using multivariate linear regression models, widowed older individuals exhibited a notably elevated risk in PP (*β* = 8.00, *p* < 0.001), fasting blood glucose levels (*β* = 0.24, *p* = 0.039), and a reduction in perceived family support (*β* = −0.66, *p* = 0.003). Nevertheless, marital status did not exhibit a substantial link with either anxiety or depressive symptoms.

Post the PSM process, a commendable balance was achieved in all covariates between the married and widowed cohorts. The outcomes of the multivariate linear regression models in this phase diverged from their pre-matching counterparts, presenting augmented beta values. Notably, being widowed was significantly correlated with diminished family support (*β* = −0.81, *p* = 0.002), elevated depressive symptoms (*β* = 1.04, *p* = 0.043), increased PP (*β* = 8.90, *p* < 0.001), and heightened fasting blood glucose levels (*β* = 3.22, *p* = 0.027).

For a comprehensive understanding of how socio-demographic covariates impact the primary outcome variables, kindly refer to the Supplementary material provided.

## Discussion

In our study focusing on older individuals in Chinese nursing homes, we elucidated the profound impact of widowhood on PP, fasting blood glucose, and mental health. Through the adept application of PSM analysis, we effectively controlled for confounding factors. Our findings were striking: widowhood considerably influenced mental health, manifesting as decreased family support (*β* = −0.81, *p* = 0.002) and heightened depressive symptoms (*β* = 1.04, *p* = 0.043). Physiologically, it correlated with increased PP (*β* = 8.90, *p* < 0.001) and elevated fasting blood glucose levels (*β* = 3.22, *p* = 0.027). The meticulous contrast between pre- and post-matching phases underscored the efficacy of PSM in ensuring covariate balance, bolstering the credibility of our results.

### Our study sheds light on the impact of widowhood on mental health outcomes

We identified a notable link between widowhood and decreased family support among older individuals in nursing homes, though no significant relationship with anxiety symptoms emerged. This association emphasized the possible social and emotional challenges confronting widowed seniors, and this trend persisted in both pre-PSM and post-PSM analyses. Given the pivotal role of family in Chinese culture, a spouse’s loss can drastically alter the family support dynamics, subsequently affecting the individual’s well-being (31). Echoing prior studies (31–33), our data demonstrated a significant correlation between depressive symptoms and widowhood. It’s apparent that seniors living alone often lack regular interactions with family or friends, potentially escalating feelings of isolation and subsequent depression (34, 35).

While many studies highlight widowhood’s impact on anxiety (36–38), our findings aligned with alternative research, such as Sonja’s study in Zadar, suggesting no direct link between widowhood and anxiety (39). This divergence can arise from multiple factors, including the widowhood duration considered. The emotional repercussions and coping strategies may evolve with time after a spouse’s loss, affecting the onset of anxiety symptoms (40). To deepen our understanding, future investigations should consider the timeline of widowhood in relation to mental health trajectories.

### Our study sheds light on the impact of widowhood on adverse physiological changes

Our findings indicated a strong link between widowhood and elevated PP in older adults nursing home residents (*β* = 8.90, *p* < 0.001). While prior studies have extensively studied the widowhood-hypertension connection with inconclusive results, the focus on PP has been limited. Liu’s research has highlighted widowhood as a significant hypertension risk in older Chinese individuals (41). Conversely, Randa’s study suggests that divorced or separated women have lower blood pressure compared to married women (40). Our study, pointing to a marked rise in PP in widowed individuals, echoed sentiments that emotional distress and lifestyle upheavals linked with widowhood can lead to cardiovascular complications (42). This reiterates the necessity for cardiovascular monitoring and interventions tailored for the widowed older adults.

On fasting blood glucose levels, we found a considerable association between widowhood and elevated glucose levels. This finding aligned with the idea of widowhood being linked to prediabetes (43). Potential dietary shifts, reduced physical activity, and heightened stress commonly experienced post-widowhood may drive metabolic disruptions (43, 44). Significantly, post-PSM linear regression revealed a heightened effect coefficient relative to pre-PSM analysis. By adeptly controlling confounding variables like age, sex, and BMI through PSM, we could precisely gauge the real impact of widowhood on fasting blood glucose levels while maintaining covariate equilibrium. This discovery stressed the imperative for interventions focused on diabetes management and prevention in widowed seniors.

### PSM analysis techniques show effective control for confounding factors

Our study underscored the importance of PSM as an instrumental technique for managing covariate characteristics. By leveraging PSM, we enhanced the comparability between the married and widowed older residents in Chinese nursing homes, especially in relation to baseline socio-demographic attributes. Prior literature has emphasized the influence of such socio-demographic variables on the widowhood-health nexus in the older adults (45, 46). For instance, studies have indicated that widowed men exhibit higher depression levels than their female counterparts (45), while women seem to better acclimate to singlehood (46).

The ‘marriage resource model’ posits that marriage endows individuals with economic, social, and psychological benefits, which collectively uplift health and extend well-being (47, 48). This crucial facet, however, has often been overlooked in previous investigations. In our analysis, the initial absolute standardized mean difference for basic socio-demographic characteristics stood at 0.533. But with PSM application, this difference substantially narrowed to 0.016, signifying a notable covariate balance in our post-matching samples. The subsequent *χ*^2^ and *T* values, which assessed socio-demographic attributes based on marital status pre- and post-matching, reinforced the efficacy of our PSM application in achieving this balance. This enhanced comparability allowed for a refined assessment in our linear regression models, where we observed distinct coefficients and beta values before and after matching. In essence, integrating PSM in observational research amplified statistical precision, bolstering the credibility and robustness of the derived conclusions.

There are several limitations to consider in this study. First and foremost, the cross-sectional nature of our data hindered the determination of causal links between the variables. To bolster our findings, subsequent studies should lean towards longitudinal designs, providing a more nuanced lens into the temporal relationships between variables. Second, while the use of PSM mitigated the influence of several socio-demographic variables, it’s crucial to recognize the potential existence of unmeasured confounders or residual confounding. Such factors might still wield influence over the relationships observed. Third, by relying on self-reported data, we inadvertently introduce the potential for biases, such as those stemming from social desirability or recall inaccuracies. Incorporating objective measures or gleaning data from multiple corroborative sources could elevate the credibility and robustness of findings in future research.

## Conclusion

The findings of this study underscored the adverse effects of widowhood on PP, fasting blood glucose, and mental health among older adults individuals in Chinese nursing homes. Compared to their married peers, widowed participants demonstrated diminished family support, an elevated risk of depressive symptoms, heightened PP, and increased fasting blood glucose levels. The application of PSM analysis bolstered the robustness of these findings by adeptly mitigating potential confounding variables.

These results accentuated the pressing need for bespoke interventions and support mechanisms designed to counteract the detrimental health outcomes tied to widowhood in the older adults. Such interventions should extend beyond merely addressing physiological repercussions and delving into the psychological and emotional ramifications of losing a spouse. As we advance, longitudinal studies become paramount to discern the prolonged influences of widowhood on diverse health facets. By grasping the intricate dynamics of these factors, policymakers, medical professionals, and caregivers can craft comprehensive strategies, fostering resilience and support for widowed seniors, thereby elevating their overall life quality.

## Data availability statement

The raw data supporting the conclusions of this article will be made available by the authors, without undue reservation.

## Ethics statement

The studies involving humans were approved by the Ethics Committee of Nantong First People's Hospital with the identification number 2023KT091. The studies were conducted in accordance with the local legislation and institutional requirements. Written informed consent for participation in this study was provided by the participants' legal guardians/next of kin.

## Author contributions

YZ: Conceptualization, Funding acquisition, Supervision, Writing – original draft. XC: Writing – review & editing, Conceptualization, Funding acquisition, Supervision. YS: Conceptualization, Funding acquisition, Supervision, Writing – original draft. SF: Conceptualization, Funding acquisition, Supervision, Writing – original draft. FW: Data curation, Investigation, Writing – review & editing. HG: Data curation, Investigation, Writing – review & editing. HJ: Data curation, Investigation, Writing – review & editing. QZ: Data curation, Investigation, Writing – review & editing. WD: Data curation, Investigation, Writing – review & editing. HL: Writing – review & editing, Formal analysis. JZ: Formal analysis, Writing – review & editing.

## References

[1] Tarakcı E Zenginler Y Kaya ME. Chronic pain, depression symptoms and daily living independency level among geriatrics in nursing home. Agri. (2015) 27:35–41. doi: 10.5505/agri.2015.14238, PMID: 25867872

[2] Chen LK. Urbanization and population aging: converging trends of demographic transitions in modern world. Arch Gerontol Geriatr. (2022) 101:104709–11. doi: 10.1016/j.archger.2022.104709 35489310

[3] Jingcheng G. International comparison and reference on home pension service mode. Soc Secur Stud. (2010) 1:29–31.

[4] Lei P Xu L Nwaru BI Long Q Wu Z. Social networks and health-related quality of life among Chinese old adults in urban areas: results from 4th National Household Health Survey. Public Health. (2016) 131:27–39. doi: 10.1016/j.puhe.2015.10.009, PMID: 26631913

[5] Fenghua C. Population aging problems and social benefit to the aged health strategies. Chin Nurs Res. (2007) 21:2152–4.

[6] Xiao HYJY Bowers B. Quality of life of nursing home residents in China: a mediation analysis. Nurs Health Sci. (2017) 19:149–56. doi: 10.1111/nhs.12288 27282918

[7] Karakaya MGBSC Ekici G Köse N Otman AS. Functional mobility, depressive symptoms, level of independence, and quality of life of the elderly living at home and in the nursing home. J Am Med Dir Assoc. (2009) 10:662–6. doi: 10.1016/j.jamda.2009.06.002, PMID: 19883891

[8] Yang C Sun X Duan W. Widowhood and life satisfaction among Chinese elderly adults: the influences of lifestyles and number of children. Front Public Health. (2021) 9:754681. doi: 10.3389/fpubh.2021.754681, PMID: 35155332 PMC8826226

[9] Pang J Xu S Wu Y. Effect of widowhood on the risk of disability among the elderly in China. Front Psych. (2023) 14:1169952. doi: 10.3389/fpsyt.2023.1169952, PMID: 37275979 PMC10232795

[10] Botev N. Population ageing in central and eastern Europe and its demographic and social context. Eur J Ageing. (2012) 9:69–79. doi: 10.1007/s10433-012-0217-9, PMID: 28804408 PMC5547418

[11] Guang-Zhou WY-X. Status of widowed elderly population in China and its development trend. Sci Res Aging. (2013) 1:4–7.

[12] Zhang Z Lin IF. Intergenerational support among widowed older adults in China. Int J Popul Stud. (2017) 3:94–109. doi: 10.18063/ijps.2017.01.003

[13] Rendall MS Weden MM Favreault MM Waldron H. The protective effect of marriage for survival: a review and update. Demography. (2011) 48:481–506. doi: 10.1007/s13524-011-0032-5, PMID: 21526396

[14] Jiang C Song H Shi J. The impact of widowhood on mental health of older adults. Geriatr Nurs. (2023) 50:38–43. doi: 10.1016/j.gerinurse.2022.12.019, PMID: 36640517

[15] Yang D Ren Z Zheng G. The impact of pension insurance types on the health of older adults in China: a study based on the 2018 CHARLS data. Front Public Health. (2023) 11:1180024. doi: 10.3389/fpubh.2023.1180024, PMID: 37333531 PMC10272461

[16] Liu X Liu F Ruan W Chen Y Qu S Wang W. Mental health status and associated contributing factors among the Hakka elderly in Fujian, China. Front Public Health. (2022) 10:928880. doi: 10.3389/fpubh.2022.1067693, PMID: 35937219 PMC9354451

[17] PRJJOM A. The consequences of divorce for adults and children. J Marriage Fam. (2010) 62:1269–87. doi: 10.1111/j.1741-3737.2000.01269.x

[18] Badhiwala JH Karmur BS Wilson JR. Propensity score matching: a powerful tool for analyzing observational nonrandomized data. Clin Spine Surg. (2021) 34:22–4. doi: 10.1097/BSD.0000000000001055 32804684

[19] Schober P Vetter TR. Propensity score matching in observational research. Anesth Analg. (2020) 130:1616–7. doi: 10.1213/ANE.0000000000004770, PMID: 32384349

[20] Liang J Hu Z Zhan C Wang Q. Using propensity score matching to balance the baseline characteristics. J Thorac Oncol. (2021) 16:e45–6. doi: 10.1016/j.jtho.2020.11.030, PMID: 34034891

[21] Pang J Liang D Wu Y. The effect of widowhood on depression of caregivers. BMC Health Serv Res. (2023) 23:722. doi: 10.1186/s12913-023-09746-4, PMID: 37400820 PMC10316613

[22] Kim HK Kim JY Kim JH Hyoung HK. Decision tree identified risk groups with high suicidal ideation in South Korea: a population-based study. Public Health Nurs. (2016) 33:99–106. doi: 10.1111/phn.12219, PMID: 26207765

[23] Mizuhara R Mitaki S Takamura M Abe S Onoda K Yamaguchi S. Pulse pressure is associated with cognitive performance in Japanese non-demented population: a cross-sectional study. BMC Neurol. (2022) 22:137. doi: 10.1186/s12883-022-02666-6, PMID: 35410174 PMC8996505

[24] de Simone G Chinali M. High pulse pressure as a marker of preclinical cardiovascular disease. Futur Cardiol. (2006) 2:165–8. doi: 10.2217/14796678.2.2.165, PMID: 19804072

[25] Balkau B Jarrett RJ Pyörälä K Eschwège E. Fasting blood glucose and risk of cardiovascular disease. Diabetes Care. (1999) 22:1385–7. doi: 10.2337/diacare.22.8.1385 10480797

[26] Jayedi A Djafarian K Rezagholizadeh F Mirzababaei A Hajimohammadi M Shab-Bidar S. Fasting blood glucose and risk of prostate cancer: a systematic review and meta-analysis of dose-response. Diabetes Metab. (2018) 44:320–7. doi: 10.1016/j.diabet.2017.09.004, PMID: 29074328

[27] Zhao X Zhang D Wu M Yang Y Xie H Li Y. Loneliness and depression symptoms among the elderly in nursing homes: a moderated mediation model of resilience and social support. Psychiatry Res. (2018) 268:143–51. doi: 10.1016/j.psychres.2018.07.011, PMID: 30025285

[28] Jongenelis K Pot AM Eisses AM Beekman AT Kluiter H Ribbe MW. Prevalence and risk indicators of depression in elderly nursing home patients: the AGED study. J Affect Disord. (2004) 83:135–42. doi: 10.1016/j.jad.2004.06.001, PMID: 15555706

[29] Procidano ME Heller K. Measures of perceived social support from friends and from family: three validation studies. Am J Community Psychol. (1983) 11:1–24. doi: 10.1007/BF00898416, PMID: 6837532

[30] Kroenke K Spitzer RL Williams JB. The PHQ-9: validity of a brief depression severity measure. J Gen Intern Med. (2001) 16:606–13. doi: 10.1046/j.1525-1497.2001.016009606.x, PMID: 11556941 PMC1495268

[31] Zhang B Li J. Gender and marital status differences in depressive symptoms among elderly adults: the roles of family support and friend support. Aging Ment Health. (2011) 15:844–54. doi: 10.1080/13607863.2011.569481, PMID: 21562986

[32] Sasson I Umberson DJ. Widowhood and depression: new light on gender differences, selection, and psychological adjustment. J Gerontol B Psychol Sci Soc Sci. (2014) 69:135–45. doi: 10.1093/geronb/gbt058, PMID: 23811294 PMC3894126

[33] Zisook S Shuchter SR. Major depression associated with widowhood. Am J Geriatr Psychiatry. (1993) 1:316–26. doi: 10.1097/00019442-199300140-00006, PMID: 28530910

[34] Srivastava S Debnath P Shri N Muhammad T. The association of widowhood and living alone with depression among older adults in India. Sci Rep. (2021) 11:21641. doi: 10.1038/s41598-021-01238-x, PMID: 34737402 PMC8568934

[35] Henning-Smith C. Quality of life and psychological distress among older adults: the role of living arrangements. J Appl Gerontol. (2016) 35:39–61. doi: 10.1177/0733464814530805, PMID: 24776792 PMC4212000

[36] You H Wang Y Xiao LD Liu L. Prevalence of and factors associated with negative psychological symptoms among elderly widows living alone in a Chinese remote sample: a cross-sectional study. Int J Environ Res Public Health. (2023) 20:264–78. doi: 10.3390/ijerph20010264, PMID: 36612585 PMC9819587

[37] van Balkom AJ Beekman AT de Beurs E Deeg DJ van Dyck R van Tilburg W. Comorbidity of the anxiety disorders in a community-based older population in the Netherlands. Acta Psychiatr Scand. (2000) 101:37–45. doi: 10.1034/j.1600-0447.2000.101001037.x, PMID: 10674949

[38] Onrust SA Cuijpers P. Mood and anxiety disorders in widowhood: a systematic review. Aging Ment Health. (2006) 10:327–34. doi: 10.1080/13607860600638529, PMID: 16798624

[39] Šare S Ljubičić M Gusar I Čanović S Konjevoda S. Self-esteem, anxiety, and depression in older people in nursing homes. Healthcare (Basel, Switzerland). (2021) 9:1035–47. doi: 10.3390/healthcare9081035 34442172 PMC8392518

[40] Kutob RM Yuan NP Wertheim BC Sbarra DA Loucks EB Nassir R. Relationship between marital transitions, health behaviors, and health indicators of postmenopausal women: results from the Women's Health Initiative. J Women's Health (2002). 2017;26:313–320, doi: 10.1089/jwh.2016.5925 PMC5397241 28072926

[41] Liu JL Guo HJ Wang Q Chen ZX Yu YK Liu XX. Status and influencing factors of hypertension in the elderly aged 60 and above in Mianyang. Zhongguo Yi Xue Ke Xue Yuan Xue Bao. (2022) 44:802–8. doi: 10.3881/j.issn.1000-503X.14695, PMID: 36325777

[42] Choi SW Rhee JA Shin JH Shin MH. The comparison of health behaviors between widowed women and married women in Jeollanamdo Province, Korea. J Prev Med Public Health. (2008) 41:272–8. doi: 10.3961/jpmph.2008.41.4.272, PMID: 18664734

[43] Assaad Khalil SH Megallaa MH Rohoma KH Ismael H AbouSeif M Kharboush I. Prevalence of type 2 diabetes mellitus in a sample of the adult population of Alexandria, Egypt. Diabetes Res Clin Pract. (2018) 144:63–73. doi: 10.1016/j.diabres.2018.07.025, PMID: 30056190

[44] Ding D Gale J Bauman A Phongsavan P Nguyen B. Effects of divorce and widowhood on subsequent health behaviours and outcomes in a sample of middle-aged and older Australian adults. Sci Rep. (2021) 11:15237. doi: 10.1038/s41598-021-93210-y, PMID: 34341364 PMC8328969

[45] Yu J Kahana E Kahana B Han C. Older men hard-hit in transition to widowhood: a 10 year longitudinal study. Innov Aging. (2018) 2:470. doi: 10.1093/geroni/igy023.1757

[46] Barry A Heale R Pilon R Lavoie AM. The meaning of home for ageing women living alone: an evolutionary concept analysis. Health Soc Care Community. (2018) 26:e337–44. doi: 10.1111/hsc.12470, PMID: 28675920

[47] Zhang Z Li LW Xu H Liu J. Does widowhood affect cognitive function among Chinese older adults? SSM Popul Health. (2019) 7:100329. doi: 10.1016/j.ssmph.2018.100329, PMID: 30581964 PMC6293047

[48] Liu H Umberson DJ. The times they are a changin': marital status and health differentials from 1972 to 2003. J Health Soc Behav. (2008) 49:239–53. doi: 10.1177/002214650804900301, PMID: 18771061 PMC3150568

