# Supplementary material for: Relationship of widowhood with pulse pressure, fasting blood glucose, and mental health in older adults: a propensity matching score analysis
Source: Front Public Health. 2023 Oct 25;11:1257133. doi: 10.3389/fpubh.2023.1257133 (PMC10634533; doi:10.3389/fpubh.2023.1257133)
Supplement: Supplementary file 1 [file Table_1.DOCX]

**Supplementary materials**

The content of the study focuses on the detailed results of the impact of widowhood on pulse pressure, fasting blood glucose, and mental health in elderly adults. This analysis was conducted both before and after employing Propensity Score Matching (PSM), and it incorporates comprehensive demographic characteristics.

**Pre-matching:**

| Outcomes-family support | variables | β (95%CI) | P |
| --- | --- | --- | --- |
|  | Age | 0.004(-0.025,0.033) | 0.775 |
|  | Sex (1=male) | 0.426(0.005,0.847) | 0.048 |
|  | Residence (1=Urban) | 0.454(-0.195,1.1028) | 0.172 |
|  | Education level (1=Bachelor’s degree or above) | 0.365(-0.135,0.865) | 0.154 |
|  | Income | 0.297(0.142,0.452) | <0.001 |
|  | BMI | 0.037(-0.0296,0.104) | 0.270 |
|  | Marital status (1=widowhood) | -0.622(-1.089,-0.235) | 0.003 |
| Outcomes-anxiety symptoms |  |  |  |
|  | Age | 0.001(-0.038,0.040) | 0.959 |
|  | Sex (1=male) | -0.294(-0.868,0.280) | 0.317 |
|  | Residence (1=Urban) | 0.694(-0.192,1.580) | 0.125 |
|  | Education level (1=Bachelor’s degree or above) | 0.119(-0.563,0.801) | 0.732 |
|  | Income | -0.044(-0.256,0.168) | 0.683 |
|  | BMI | -0.087(-0.177,0.003) | 0.059 |
|  | Marital status (1=widowhood) | -0.168(-0.748,0.412) | 0.571 |
| Outcomes-depressive symptoms |  |  |  |
|  | Age | -0.029(-0.090,0.032) | 0.348 |
|  | Sex (1=male) | 0.010(-0.872,0.892) | 0.982 |
|  | Residence (1=Urban) | 0.185(-1.173,1.543) | 0.79 |
|  | Education level (1=Bachelor’s degree or above) | -0.096(-1.143,0.951) | 0.857 |
|  | Income | -0.132(-0.455,0.191) | 0.426 |
|  | BMI | -0.082(-0.219,0.055) | 0.245 |
|  | Marital status (1=widowhood) | 0.851(-0.041,1.743) | 0.062 |
| Outcomes- fasting blood glucose |  |  |  |
|  | Age | -0.021(-0.037,-0.005) | 0.008 |
|  | Sex (1=male) | -0.23(-0.453,-0.007) | 0.044 |
|  | Residence (1=Urban) | -0.155(-0.500,0.190) | 0.38 |
|  | Education level (1=Bachelor’s degree or above) | -0.314(-0.579,-0.049) | 0.021 |
|  | Income | -0.037(-0.119,0.045) | 0.373 |
|  | BMI | 0.033(-0.002,0.068) | 0.065 |
|  | Marital status (1=widowhood) | 0.238(0.013,0.463) | 0.039 |
| Outcomes- pulse pressure |  |  |  |
|  | Age | -0.142(-0.385,0.101) | 0.250 |
|  | Sex (1=male) | -3.140(-6.644,0.364) | 0.080 |
|  | Residence (1=Urban) | 3.019(-2.387,8.425) | 0.274 |
|  | Education level (1=Bachelor’s degree or above) | -1.904(-6.069,2.261) | 0.371 |
|  | Income | 2.11 (0.824,3.404) | 0.001 |
|  | BMI | 0.394(-0.155,0.943) | 0.159 |
|  | Marital status (1=widowhood) | 8.000(4.452,11.548) | <0.001 |

**Post-matching:**

| Outcomes-family support | variables | β (95%CI) | P |
| --- | --- | --- | --- |
|  | Age | 0.006(-0.037,0.049) | 0.766 |
|  | Sex (1=male) | 0.547(-0.009,1.103) | 0.055 |
|  | Residence (1=Urban) | 0.306(-0.470,1.082) | 0.441 |
|  | Education level (1=Bachelor’s degree or above) | 0.014(-0.646,0.674) | 0.967 |
|  | Income | 0.320(-0.122,0.517) | 0.002 |
|  | BMI | 0.018(-0.072,0.108) | 0.69 |
|  | Marital status (1=widowhood) | -0.811(-1.330,-0.292) | 0.002 |
| Outcomes-anxiety symptoms |  |  |  |
|  | Age | 0.003(-0.049,0.055) | 0.919 |
|  | Sex (1=male) | -0.128(-0.829,0.573) | 0.721 |
|  | Residence (1=Urban) | 0.399(-0.581,1.379) | 0.426 |
|  | Education level (1=Bachelor’s degree or above) | 0.532(-0.301,1.365) | 0.213 |
|  | Income | -0.066(-0.316,0.184) | 0.607 |
|  | BMI | -0.058(-0.171,0.055) | 0.32 |
|  | Marital status (1=widowhood) | -0.070(-0.727,0.587) | 0.834 |
| Outcomes-depressive symptoms |  |  |  |
|  | Age | -0.052(-0.134,0.030) | 0.217 |
|  | Sex (1=male) | 0.311(-0.757,1.379) | 0.569 |
|  | Residence (1=Urban) | 0.211(-1.282,1.704) | 0.782 |
|  | Education level (1=Bachelor’s degree or above) | 0.233(-1.037,1.503) | 0.719 |
|  | Income | -0.066(-0.448,0.316) | 0.734 |
|  | BMI | -0.079(-0.253,0.095) | 0.371 |
|  | Marital status (1=widowhood) | 1.037(0.035,2.039) | 0.043 |
| Outcomes- fasting blood glucose |  |  |  |
|  | Age | -0.012(-0.031,0.007) | 0.229 |
|  | Sex (1=male) | -0.303(-0.561,-0.04) | 0.023 |
|  | Residence (1=Urban) | -0.043(-0.405,0.319) | 0.816 |
|  | Education level (1=Bachelor’s degree or above) | -0.236(-0.543,0.071) | 0.135 |
|  | Income | -0.049(-0.141,0.043) | 0.3 |
|  | BMI | 0.022(-0.019,0.063) | 0.306 |
|  | Marital status (1=widowhood) | 0.276(0.033,0.519) | 0.027 |
| Outcomes- pulse pressure |  |  |  |
|  | Age | -0.129(-0.483,0.225) | 0.478 |
|  | Sex (1=male) | -4.530(-9.185,0.125) | 0.058 |
|  | Residence (1=Urban) | 3.533(-2.974,10.04) | 0.288 |
|  | Education level (1=Bachelor’s degree or above) | -3.302(-8.835,2.231) | 0.243 |
|  | Income | 1.87(-0.209,3.530) | 0.028 |
|  | BMI | 0.344(-0.412,1.100) | 0.374 |
|  | Marital status (1=widowhood) | 8.901(4.538,13.264) | <0.001 |
